# Colloid Mill-Assisted Ultrasonic-Fractional Centrifugal Purification of Low-Grade Attapulgite and Its Modification for Adsorption of Congo Red

**DOI:** 10.3390/ma17092034

**Published:** 2024-04-26

**Authors:** Xingpeng Wang, Chao Jiang, Huiyu Li, Weiliang Tian, Saeed Ahmed, Yongjun Feng

**Affiliations:** 1 State Key Laboratory of Chemical Resource Engineering, College of Chemistry, Beijing University of Chemical Technology, Beijing 100029, China; wxp_9786@163.com (X.W.); 15222443969@163.com (C.J.); 2 College of Chemistry and Chemical Engineering, Tarim University, Alar 843300, China; 120100037@taru.edu.cn; 3 Department of Chemistry, University of Chakwal, Chakwal 48800, Pakistan; saeed.ahmed@uoc.edu.pk; 4 Gansu West Attapulgite Application Research Institute, Baiyin 730900, China

**Keywords:** attapulgite, purification, colloid mill, modification, Congo red

## Abstract

Attapulgite (APT) is widely used in wastewater treatment due to its exceptional adsorption and colloidal properties, as well as its cost-effectiveness and eco-friendliness. However, low-grade APT generally limits its performance. Here, a colloid mill-assisted ultrasonic-fractional centrifugal purification method was developed to refine low-grade APT. This process successfully separated and removed impurity minerals such as quartz and dolomite from the raw ore, resulting in a refined APT purity increase from 16.9% to 60% with a specific surface area of 135.5 m^2^∙g^−1^. Further modifying of the refined APT was carried out through the hydrothermal method using varying dosages of cetyltrimethylammonium chloride (CTAC), resulting in the production of four different APT adsorbents denoted as QAPT-*n* (*n* = CTAC mole number) ranging from 0.5 to 5 mmol. Using Congo red (CR) as the target pollutant, the QAPT-5 sample exhibited the best adsorption capacity with the maximum quantity of 1652.2 mg∙g^−1^ in a neutral solution at 30 °C due to the highest surface charge (zeta potential = 8.25 mV). Moreover, the QAPT-5 pellets (~2.0 g adsorbent) shaped by the alginate-assisted molding method removed more than 96% of 200 mL aqueous solution containing 200 mg∙L^−1^ CR and maintained this efficiency in 10 adsorption–elution cycles, which exhibited the promising practical application.

## 1. Introduction

Attapulgite (APT) is one kind of natural nanoscale magnesium-aluminosilicate clay with the theoretical chemical formula Mg_5_Si_8_O_20_(HO)_2_(OH_2_)_4_∙4H_2_O [1,2,3]. Here, H_2_O, (OH_2_), and (OH) represent zeolite water, allotropic water, and tectonic water, respectively [3]. In 1940, Bradley proposed a crystal structure model for attapulgite, which consists of inversed silica-oxygen tetrahedral double chains and discontinuous octahedral sheets [1]. The chain-layer units are connected by Si-O-Si bonds to form zeolite-like pores with a size of 0.37 nm × 0.64 nm. APT exhibits a unique fibrous or rod-like microstructure with three levels: bar crystals, crystal bundles, and aggregates [4]. These unique characteristics provide APT with excellent carrier performance, colloidal performance, adsorption performance, and reinforcement performance [5]. In recent decades, research on APT has primarily focused on modifying high-purity APT to achieved better effects in pollution abatement [6], soil remediation [7], modern agriculture [8], chemical additives [9,10], biomedicine materials [11,12,13], energy materials [14], electromagnetic absorption materials [15], building materials [16], and other industries.

China possesses more than 60% of the world’s reserves of APT, with Gansu Province ranking at the top within the country for potential APT reserves [17]. APT in Gansu Province is a product of the sedimentary metamorphism of salt-lake phases [18]. Essentially, natural APT exists in the form of crystal bundles and aggregates alongside various associated minerals like muscovite, chlorite, dolomite, and feldspar [19]. Historically, the purification and dispersion of low-grade APT have been primarily addressed through the modification of APT by different physical or chemical methods. Traditional techniques such as grinding [20] and extrusion [21] are typically used for processing high-grade APT to enhance its adsorption and colloidal properties. In a departure from these conventional methods, high-pressure homogenization technology has been utilized to disaggregate APT crystal bundles without damaging the rods for large-scale processing [22]. Furthermore, ultrasound has been employed to break up large agglomerates of APT rods into smaller crystal bundles or rod crystals [23]. The integration of processes such as extrusion, slurry, surface modification, and high-pressure homogenization has proven successful in efficiently dissociating APT crystal bundles on an industrial scale [24]. Nonetheless, while these methods effectively reduce the size of APT particles and enhance the dispersion of rod crystals, they are more suited for APT of higher purity, with less pronounced effects on lower-grade APT. Colloid milling has been widely used in the production of functional nanomaterials like layered double hydroxides [25], barium sulfate [26], and boehmite [27] in both laboratory and industrial settings. Therefore, a combination of colloid milling and ultrasound techniques may offer a viable approach to enhancing the purity of low-grade APT.

Generally, APT has been widely investigated for cation removal, and it is limited for anions due to its surface negative charge. Hence, natural APT is commonly utilized for the adsorption of cationic dyes, such as methylene blue [28], methyl violet [29], and methyl red [30], while exhibiting limited adsorption capabilities for anionic dyes. To overcome this limitation, surfactant modification through adsorption or ion exchange has been commonly utilized in clay minerals to enhance their adsorption properties. The quaternary ammonium salt modification can alter the surface charge characteristics of APT, thereby converting its negatively charged surface into a positively charged adsorbent material suitable for the adsorption of anionic dye. In this study, we propose an innovative approach for refining low-grade APT and producing organic APT using cetyltrimethylammonium chloride (CTAC) for the adsorption of the anionic dye Congo red (CR). Our objectives include investigating the efficiency of CR removal by the modified adsorbents, assessing the recyclability of modified APT pellets for CR adsorption, and examining the adsorption mechanism of the modified APT towards the CR.

## 2. Materials and Methods

### 2.1. Materials

The main raw material, APT, was obtained from the Gansu Western Attapulgite Research and Application Institute in Baiyin, China, and sieved over a 200-mesh sieve. The composition of APT varies depending on the difference in natural attapulgite deposits. The APT (purity is 16.9%) comprised mainly 48.74%, 16.63%, 14.69%, and 5.64% of SiO_2_, Fe_2_O_3_, Al_2_O_3_, and MgO, respectively, presented in Table 1. Tetrasodium pyrophosphate (Na_4_P_2_O_7_) and cetyltrimethylammonium chloride (CTAC) were purchased from Shanghai Aladdin Biochemical Technology Co., Ltd. (Shanghai, China). Congo red (CR) was obtained from Xilong Scientific Co., Ltd. (Shantou, China). All the used reagents were analytical grade and without further purification. Deionized water was used throughout.

### 2.2. Methods

#### 2.2.1. Purification of Low-Grade Attapulgite

Firstly, 4.5 g of sodium pyrophosphate (Na_4_P_2_O_7_) dispersant was dissolved in 1.5 L deionized water to prepare the dispersant solution. Subsequently, 150.0 g of APT powder was introduced into the solution and uniformly dispersed through continuous mechanical stirring for 30 min. The resulting homogeneous slurry was passed through a colloid mill three times at 3000 rpm/min, maintaining a slit width of 0.1 mm. Following this, the APT slurry was sonicated for 1 h with mechanical agitation. After the slurry stood for 2 h, fractional centrifugation was conducted in two stages. The first stage involved centrifuging at 2800 rpm for 30 min, followed by the second stage of separation at 3800 rpm for 5 min. To break the emulsion at the beginning of the second centrifugation stage, a specific amount of ethanol or dilute acid was added in the suspension. Finally, the precipitate was dried at 70 °C for 12 h and then ground for further use. The obtained sample was designated as TAPT.

#### 2.2.2. Preparation of Modified Attapulgite

A specific amount of quaternary ammonium salt modifier CTAC was dissolved in 50 mL of deionized water. The solution was then ultrasonically dispersed and magnetically stirred until it was uniformly mixed. Subsequently, 5.0 g of TAPT was added to the solution while continuously stirring. After 30 min of stirring, the mixture was transferred to a 100 mL polytetrafluoroethylene reactor, and the reaction was carried out at 180 °C for 12 h. Once the reaction was completed, the slurry was allowed to cool down to room temperature before being centrifuged. The resulting material was washed with hot water until no Cl^−^ could be detected. After drying in an oven at 60 °C, quaternary ammonium salt-modified attapulgite was obtained and ground for further use. The molar mass values of CTAC were 0.5, 1.0, 3.0, and 5.0 mmol, and the samples were named QAPT-0.5, QAPT-1.0, QAPT-3.0, and QAPT-5.0 according to the molar mass of CTAC, respectively.

#### 2.2.3. Preparation of Attapulgite Pellets

The QAPT powder was molded into attapulgite pellets using an alginate-assisted molding method [31]. Initially, 0.1 g of sodium alginate (SA) was dissolved in 7.0 mL of deionized water through magnetic stirring for 2 h at 50 °C, resulting in a uniform and clear solution. Subsequently, 0.9 g of the QAPT-5 powder was dispersed in the SA solution under vigorous stirring for another 2 h, yielding the SA/QAPT sol. The SA/QAPT sol was then carefully dripped into a 50 mL solution of 3% (w.t.) CaCl_2_ using a 10 mL syringe and a No. 5 needle. During this process, the droplets of SA/QAPT sol made contact with the CaCl_2_ solution, forming gel pellets. After completion, the gel pellets were left in the CaCl_2_ solution for 12 h, followed by washing with deionized water until no Cl^−^ was detected in the rinse solution with 0.1 mol∙L^−1^ AgNO_3_ solution. The pellets were then dried at 60 °C for 12 h. The as-prepared attapulgite pellets, with diameters ranging from 2 to 3 mm, were utilized in the construction of the Congo red adsorption column.

#### 2.2.4. Congo Red Adsorption Capacities of QAPT

The adsorption kinetics experiments for Congo red (CR) on attapulgite adsorbents were conducted by dispersing 25.0 mg of attapulgite in 100.0 mL of 200 mg∙L^−1^ CR aqueous solution. The resulting suspension was then shaken in a shaker at 20 °C for 24 h. Afterwards, the samples were filtered through a 0.45 µm needle filter and the residual CR concentration was promptly measured using a UV-vis spectrophotometer (Shimadzu UV-2600, Kyoto, Japan) at *λ_max_* = 497 nm at specific time intervals.

To determine the adsorption isotherm of CR, 25.0 mg of adsorbent was initially mixed with varying concentrations of CR solution (ranging from 200 to 1000 mg∙L^−1^) in Erlenmeyer flasks, each containing 100.0 mL of solution. These flasks were then shaken in a thermostatic shaker for 24 h, and the equilibrium concentration of the adsorbent was measured by UV-vis spectroscopy.

All adsorption experiments were conducted in triplicate, and the average values were utilized for analysis in this work.

#### 2.2.5. Adsorbents’ Characterization

The chemical analysis of the samples was performed with a Shimadzu XRF-1800 spectrometer (Kyoto, Japan). The crystalline structure was determined through powder X-ray diffraction analysis (PXRD) conducted on a Shimadzu XRD-6000 instrument (Kyoto, Japan) with Cu Kα radiation (*λ* = 1.5418 Å), scanning at a rate of 5°/2θ min^−1^ from 3 to 70°/2θ. Morphological characteristics were examined using a Zeiss Supra 55 scanning electron microscope (SEM, Jena, Germany) at 20 kV. Specific surface area and pore properties were calculated based on N_2_ adsorption–desorption isotherms recorded on a Quantachrome 3QDS-MP-30 (Boynton Beach, FL, USA) at 77 K. Fourier transform infrared (FT-IR) spectra were collected on a Bruker Vector 22 spectrophotometer (Karlsruhe, Germany) employing the KBr pellet technique (sample/KBr ratio of 1/99 by mass) across the range of 4000 to 400 cm^−1^, with a resolution of 2 cm^−1^. The TGA analysis of the nanoparticles was performed with a Shimadzu DTG-60AH system (Kyoto, Japan), with heating at a rate of 10 °C/min under an N_2_ atmosphere with a flow rate of 50 mL/min. A 0.5% (w.t.) sample suspension was prepared and dispersed through sonication for 10 min to evaluate the zeta potential of the attapulgite samples on a Malvern ZEN 3600 Instrument (Malvern Panalytical, Almelo, The Netherlands). X-ray photoelectron spectroscopy (XPS) spectra were examined utilizing the Kratos AXIS SUPRA spectrometer (Manchester, UK) with Al Kα radiation (1486.6 eV). The C 1s signal at 284.8 eV was utilized to calibrate the binding energy scale. Deconvolution of the XPS results was performed using the XPSPEAK41 software (6.00.8450) with a mixed function of 20% Lorentz and 80% Gauss.

#### 2.2.6. Statistical Method

The quantity of CR adsorbed by attapulgite at time *t*, $q_{t}$ (mg∙g^−1^), was calculated using the following equation:

(1) $$q_{t}=\frac{\left(C_{0}-C_{t}\right)\times V}{m},$$

where $C_{0}$ (mg∙L^−1^) signifies the initial concentration of the CR solution, $C_{t}$ (mg∙L^−1^) represents the CR concentration at time *t*; while *V* (L) and *m* (g) refer to the volume of the CR solution and the mass of the adsorbent added, respectively.

To delve deeper into the adsorption behavior of CTAC-modified attapulgite samples on CR, three typical adsorption kinetic models were employed: the pseudo-first-order (PFO) kinetic equation (Equation (2)) [32], the pseudo-second-order (PSO) equation (Equation (3)) [33], and the intra-particle diffusion equation (Equation (4)) [34] for kinetic analyses of the experimental data.

(2) $$q_{t}=q_{e}\left(1-e^{-k_{1}t}\right),$$

(3) $$q_{t}=\frac{q_{e}^{2}k_{2}t}{{1+k}_{2}q_{e}t},$$

(4) $$q_{t}=k_{p}\sqrt{t}+C,$$

where $q_{t}$ (mg∙g^−1^) and $q_{e}$ (mg∙g^−1^) are the amounts of CR uptake per mass of the APT sample at any time *t* (min) and at equilibrium, respectively; $k_{1}$ (min^−1^), $k_{2}$ (g∙mg^−1^∙min^−1^), and $k_{p}$ (mg∙g^−1^∙min^−1/2^) are the rate constants for the PFO equation, PSO equation, and intra-particle diffusion equation, respectively; and *C* (mg∙g^−1^) is a constant associated with the boundary layer thickness.

The adsorption isotherm data of samples to CR in water were subjected to fitting and analysis using the Langmuir model (Equation (5)) [35] and Freundlich model (Equation (6)) [36].

(5) $$q_{e}=\frac{q_{max}K_{L}C_{e}}{1+K_{L}C_{e}},$$

(6) $$q_{e}=K_{F}C_{e}^{n},$$

where $q_{e}$ (mg∙g^−1^) and $q_{max}$ (mg∙g^−1^) represent the equilibrium and maximum adsorption of CR by APT samples, respectively; *C_e_* denotes the equilibrium concentration of CR in the solution; *K_L_* and *K_F_* are Langmuir and Freundlich equilibrium constants, respectively; and *n* signifies the trend of adsorption.

## 3. Results

### 3.1. Composition, Structure, and Morphology of TAPT

**Figure 1:**
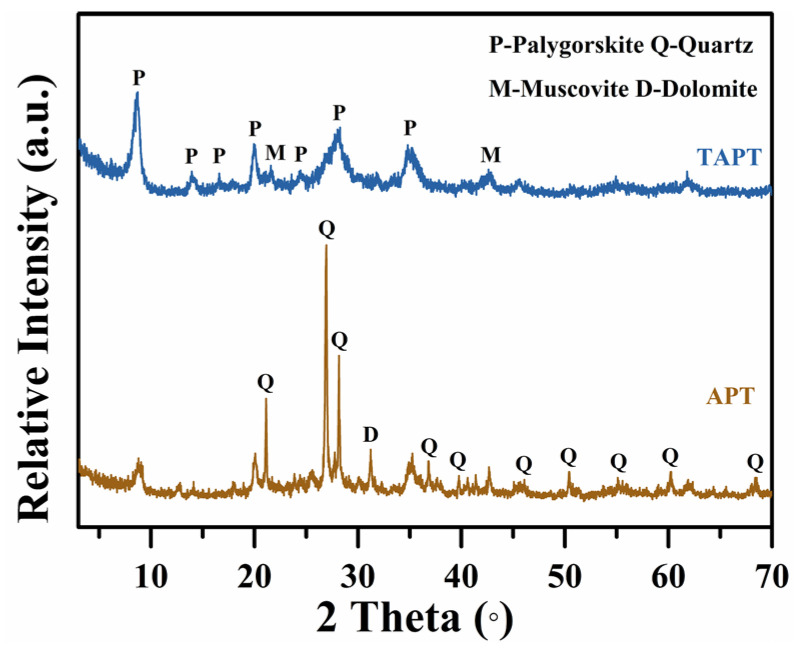
Powder X-ray diffraction patterns of APT and TAPT.

Figure 1 shows the XRD patterns of APT and TAPT. Here, the diffraction peaks of APT at 2θ = 8.4°, 14.1°, 34.9°, and 35.3°are ascribed to the characteristic reflections of the (110), (200), (102), and (161) crystalline planes of APT, respectively [37]. In addition, other reflections are for quartz at 2θ = 21.1°, 26.9°, 28.2°, 40.6°, 42.7°, 46.1°, 50.4°, 55.2°, 60.3°, 64.3°, and 68.4° (JCPDS PDF card# 85-0865, 83-0542); dolomite at 2θ = 31.2° (JCPDS PDF card# 36-0426); and muscovite at 2θ = 23.9° and 42.4° (JCPDS PDF card# 89-6216), indicating the presence of associated minerals in the APT. After the purification, the characteristic peaks of quartz and dolomite almost disappear, and the diffraction intensity for attapulgite strengthens greatly, indicating that most of associated minerals were removed effectively. This result is consistent with the chemical composition of the samples listed in Table 1. The contents of CaO, K_2_O, and TiO_2_ decrease significantly, except for a slight increase in the contents of P_2_O_5_ and Na_2_O, which result from the tetrasodium pyrophosphate dispersant and the removal of impurity minerals such as quartz and dolomite. Finally, the purity of TAPT reaches ca. 60%.

**Figure 2:**
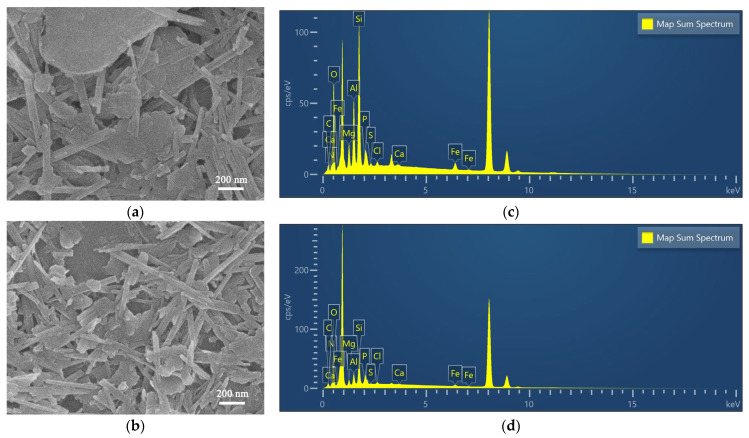
SEM images and EDX analysis for APT (**a**,**c**) and TAPT (**b**,**d**).

**Figure 3:**
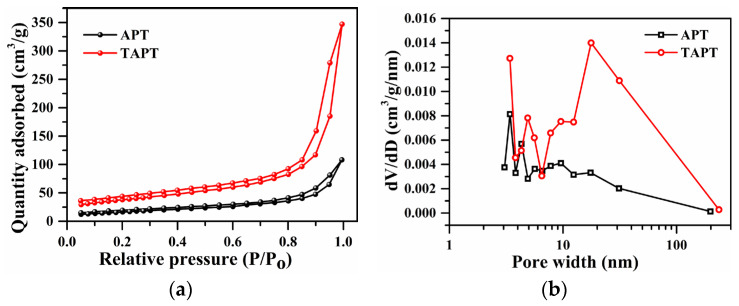
N_2_ adsorption–desorption isotherms (**a**) and pore size distribution (**b**) of APT and TAPT.

In Figure 2a,b, the rod-like crystals and sheet-like particles occur in the SEM images of APT, indicating the coexistence of attapulgite and associated minerals in APT. However, plentiful rod crystals are observed obviously in the SEM images of TAPT, indicating that the impurity minerals were almost removed. Figure 2c,d further illustrate a significant decrease in the Ca content of the refined APT, suggesting the removal of dolomite, a finding that aligns with the XRD analyses of the samples. Moreover, as a large number of impurity minerals were separated and removed by the refining process, rod crystals became looser and pore channels became more open, which aid in the increase in the specific surface area of TAPT from 60.57 to 135.5 m^2^·g^−1^ and enlarge the average pore size from 3.42 to 17.67 nm (Figure 3 and Table 2) compared with APT. The above results suggest that the colloid mill-assisted ultrasonic dispersion fractional centrifugal purification method is effective to refine attapulgite.

### 3.2. Characteristics of QAPT Samples

**Figure 4:**
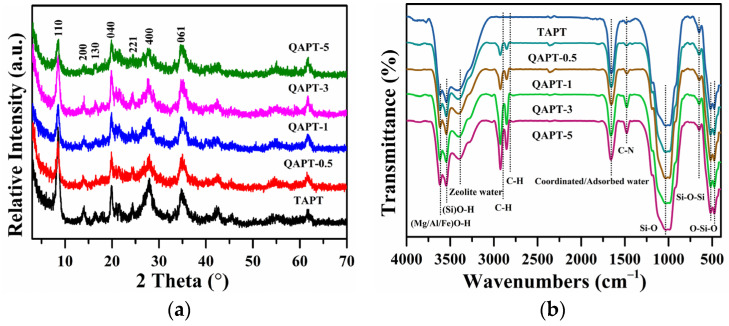
X-ray diffraction patterns (**a**) and FT-IR spectra (**b**) of QAPT samples.

Figure 4a displays the XRD patterns of the TAPT, QAPT-0.5, QAPT-1, QAPT-3, and QAPT-5 samples. Here, all of the five samples exhibit the similar characteristic diffraction peaks of the ATP, and those of quartz, muscovite, and other impurity minerals are not obvious. After the hydrothermal reaction, the intensity of the characteristic diffraction peaks of attapulgite slowly weakens with the increasing amount of CTAC from 0 to 5 mmol, especially the (110) crystal plane diffraction peak. This may indicate that the strong interaction between the CTAC modifier and attapulgite mainly occurs in the direction of the (110) crystal plane of the attapulgite, and the CTAC is only encapsulated or grafted on the surface of the attapulgite and does not damage the crystal structure of the attapulgite.

Fourier transform infrared spectroscopy (FT-IR) confirmed the presence of organic components in the QAPT adsorbents. In Figure 4b, the FT-IR spectra of the QATP adsorbents show symmetric and antisymmetric telescopic vibration peaks of -CH_2_ for the absorption peaks at 2926 and 2851 cm^−1^, respectively, and the deformation vibration peaks of the -CH_2_ group for the absorption peak at 1476 cm^−1^. Meanwhile, the intensity of these absorption peaks is enhanced with the dosage increase in the modifier CTAC, and the intensity of this absorption peak no longer increased when the CTAC dosage was 5 mmol. Before and after the hydrothermal reaction, the characteristic absorption peaks of attapulgite do not change significantly. Among them, the absorption peaks at 3619, 3544, and 3404 cm^−1^ are the telescopic vibrations of (Mg/Al/Fe)O-H, (Si)O-H, and zeolite water, respectively; the absorption peak at 1654 cm^−1^ is the bending vibration peak of coordination water or adsorption water; the absorption peaks at 1031 and 652 cm^−1^ are attributed to the stretching vibrations of Si-O and inverted tetrahedral skeleton SiO_4_, respectively; and the absorption peaks at 517 and 474 cm^−1^ are attributed to the stretching vibrations of Si-O-Si and O-Si-O bending vibrations, respectively [37]. These results confirm the successful incorporation of CTAC into QAPT, adsorbent through electrostatic, hydrophobic, intermolecular forces, and hydrogen bonding [38].

**Figure 5:**
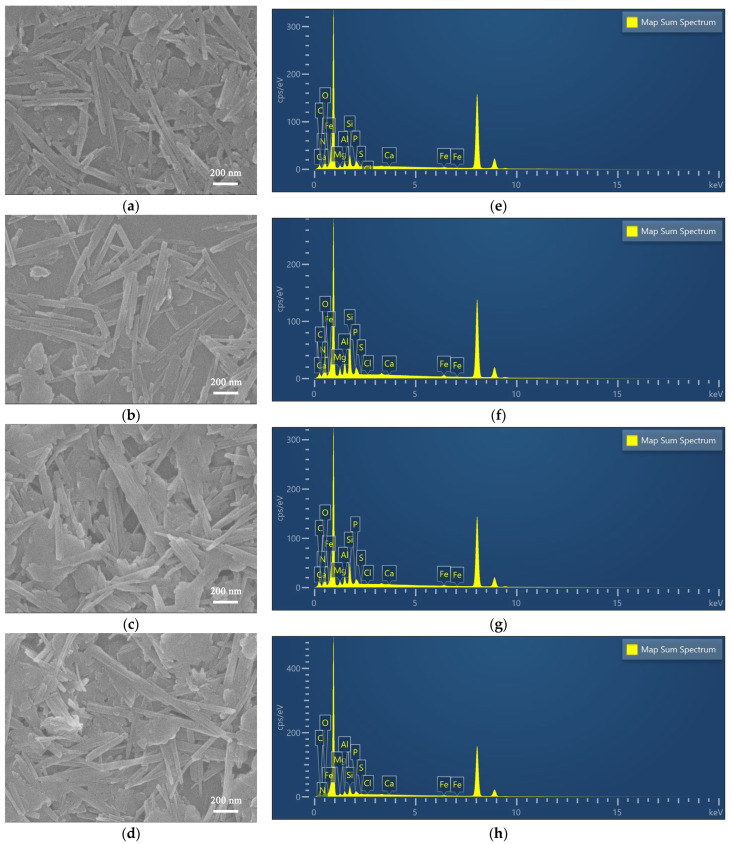
SEM images and EDX analysis for QAPT-0.5 (**a**,**e**), QAPT-1 (**b**,**f**), QAPT-3 (**c**,**g**), and QAPT-5 (**d**,**h**).

Figure 5 displays the SEM images of the TAPT, QAPT-0.5, QAPT-1, QAPT-3, and QAPT-5 samples. The SEM characterization reveals significant changes in sample morphology with increasing CTAC molarity. For instance, when the CTAC molarity is 0, sample TAPT exhibits a disordered rod stacking structure. However, in samples QAPT-0.5 and QAPT-1, the rod crystals become dispersed and orderly, leading to a reduction in the stacked firewood-like structure. Furthermore, as the CTAC molarity continues to increase to 3–5 mmol, the rod crystals in the QAPT samples start to aggregate into bundles, and even larger aggregates of rod crystals appear. These observations can be attributed to the negative charge on the surface of TAPT, which attracts the positively charged CTAC. Consequently, CTAC molecules wrap around the TAPT surface. With an increase in CTAC content, more positive quaternary ammonium salt ions bind to the surface of the one-dimensional nanorod crystals in TAPT, consequently leading to changes in the carbon and hydrogen contents within the samples (Figure 5e–h). This enhances the attraction between neighboring rod crystals, resulting in their initial agglomeration, the formation of more rod crystal bundles, and even the emergence of aggregates of rod crystals [39].

**Figure 6:**
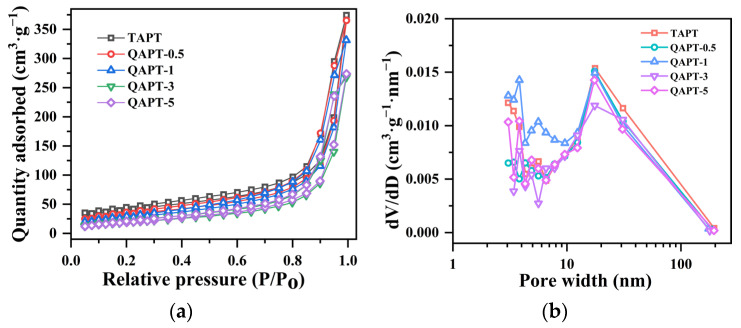
N_2_ adsorption–desorption isotherms (**a**) and pore size distribution (**b**) of TAPT and QAPT adsorbents.

Figure 6 illustrates the N_2_ adsorption and desorption curves and pore size distributions at 77 K for samples TAPT, QAPT-0.5, QAPT-1, QAPT-3, and QAPT-5. In Figure 6a, all the samples display the type IVa isotherm, indicating that the interaction between N_2_ molecules and adsorbents is significant. The hysteresis loop corresponds to the typical H3-type, suggesting the presence of slit pores formed by the stacking of lamellar particles [40,41]. These findings are consistent with the observations shown in Figure 2b and Figure 5a–d. Figure 6b shows that the pore size distributions of all five samples exhibit three distinct regions: 2~4 nm, 4~10 nm, and 10~200 nm. Moreover, the pore volume distributions within the 10~200 nm range are similar among all the samples. However, notable differences arise in the 2~4 nm and 4~10 nm ranges: an increase in the CTAC molar amount, especially when the CTAC molar amount is ≥3 mmol, leads to a significant decrease in pore sizes below 4 nm. Additionally, the QAPT-5 sample exhibits a higher number of pores ranging from 10 to 200 nm compared to the QAPT-3 sample. Table 2 summarizes the results of the BET analysis for TAPT, QAPT-0.5, QAPT-1, QAPT-3, and QAPT-5. As the CTAC molar amount increases from 0 to 5 mmol, the specific surface area gradually decreases from 135.5 m^2^∙g^−1^ to 71.29 m^2^∙g^−1^. Meanwhile, the pore volume initially increases (from 0.50 cm^3^∙g^−1^ to 0.55 cm^3^∙g^−1^) and then decreases (to 0.43 cm^3^∙g^−1^), and the average pore size gradually decreases from 17.67 nm to 17.43 nm. These results indicate that the specific surface area of the samples decreases, hindering the nanopore channels; however, the surface adsorption sites increase after CTAC modification.

**Figure 7:**
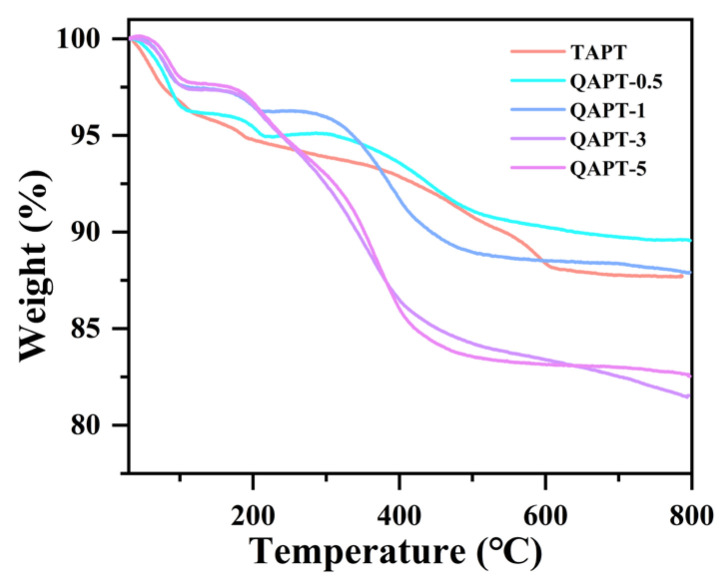
TGA curves of TAPT and QAPT adsorbents.

The effect of CTAC modifier dosage on TAPT is further demonstrated by the TGA curves of QAPT adsorbents. As depicted in Figure 7, when the molar amount of the CTAC modifier ranged from 0.5 to 1 mmol, QAPT-1 exhibited a larger mass loss compared with QAPT-0.5, but both were lower than or close to the loss mass of TAPT. On the other hand, when the molar amount of CTAC ranged from 3 to 5 mmol, both QAPT-3 and QAPT-5 displayed a higher mass loss than TAPT. This can be attributed to the successful encapsulation of CTAC on the surface of the TAPT, effectively impeding the release of adsorbed water, zeolite water, and allotropic water from the pore channels of the rod crystals. Additionally, a lower amount of modifier led to a greater mass loss in the TAPT sample in relation to QAPT-0.5 and QAPT-1. The decomposition of the organic species in QAPT adsorbents mainly occurs within the temperature range of 200–650 °C, primarily due to the oxidation of hydrocarbon and ammonium groups [42].

### 3.3. Adsorption Property of QAPT Adsorbents for Congo Red

#### 3.3.1. Adsorption Kinetics of QAPT Adsorbents for Congo Red

**Figure 8:**
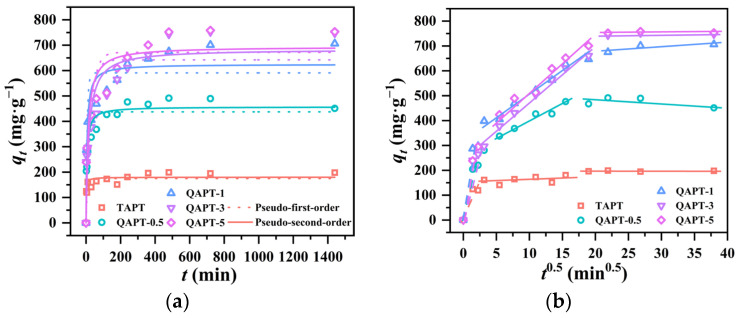
The nonlinear fitting of kinetics (**a**) and intra-particle diffusion kinetics (**b**) of adsorbents for CR.

Figure 8 presents the results of the adsorption kinetics of TAPT, QAPT-0.5, QAPT-1, QAPT-3, and QAPT-5 on CR ($C_{0}$ = 200 mg∙L^−1^, 20 °C). In Figure 8a, one can observe that TAPT, QAPT-0.5, and QAPT-1 possess larger specific surface areas with a faster adsorption equilibrium than QAPT-3 and QAPT-5. Interestingly, the equilibrium adsorption amounts of QAPT-3 and QAPT-5, with smaller specific surface areas ($q_{e}$ values of 752.4 and 758.4 mg∙g^−1^, respectively), are significantly higher than those of TAPT, QAPT-0.5, and QAPT-1 ($q_{e}$ values of 198.8, 491.3, and 706.2 mg∙g^−1^, respectively). Furthermore, while the QAPT-3 has a slightly larger specific surface area than that of QAPT-5 (72.53 and 71.29 m^2^∙g^−1^, respectively), QAPT-5 exhibits similar $q_{e}$ values and equilibrium time as QAPT-3. Additionally, despite the smaller average pore size of QAPT-5 compared with QAPT-3 (17.43 and 17.55 nm, respectively), QAPT-5 displays a faster adsorption rate. These findings suggest that a larger specific surface area enhances the adsorption rate when the modifier’s effect on the adsorption outcomes is not taken into account.

To further explore the adsorption behavior of QAPT on CR, the PFO kinetic model, PSO kinetic model, and the inter-particle diffusion model were employed for kinetic analyses of the experimental data. The results derived from fitting each model are illustrated in Figure 8a,b as well as summarized in Table 3. The results of the nonlinear fitting for CR adsorption by all samples indicate a closer alignment with the PSO kinetic model, suggesting a potential chemisorption process between the adsorbents and CR. This preference stems from the fact that the correlation coefficient (*R*^2^) of the nonlinear fitting using the PSO kinetic model is closer to 1 than that of the PFO kinetic model. Moreover, the calculated equilibrium adsorption amount ($q_{e,\;max}$) derived from the nonlinear fitting of the PSO model better matches the experimental equilibrium adsorption amount ($q_{e,\;exp}$). The magnitude of the rate constant $k_{2}$ in the PSO kinetic model signifies that the adsorption rate of CR by each sample follows the order of TAPT > QAPT-0.5 > QAPT-1 > QAPT-5 > QAPT-3. It is evident that samples with larger specific surface areas exhibit faster adsorption rates compared to samples with smaller specific surface areas. Similarly, samples with similar specific surface areas but larger pore volumes demonstrate faster adsorption rates. Therefore, sample QAPT-5 has the best adsorption performance for the CR solution.

Figure 8b depicts the fitted intra-particle diffusion model for CR adsorption by TAPT, QAPT-0.5, QAPT-1, QAPT-3, and QAPT-5, revealing that the adsorption of CR by each adsorbent manifests in three linear regions. This observation suggests that the adsorption process is governed by a multi-step mechanism involving “film diffusion”, “intra-particle diffusion”, and adsorptive attachment [43]. Notably, intra-particle diffusion emerges as the primary rate-controlling step in the CR adsorption process. Furthermore, both TAPT and QAPT-0.5 undergo shorter intra-particle diffusion processes and reach equilibrium earlier in relation to the other three samples. This expedited equilibrium attainment can be attributed to their relatively larger specific surface areas.

#### 3.3.2. Adsorption Thermodynamics of QAPT-5 for Congo Red

**Figure 9:**
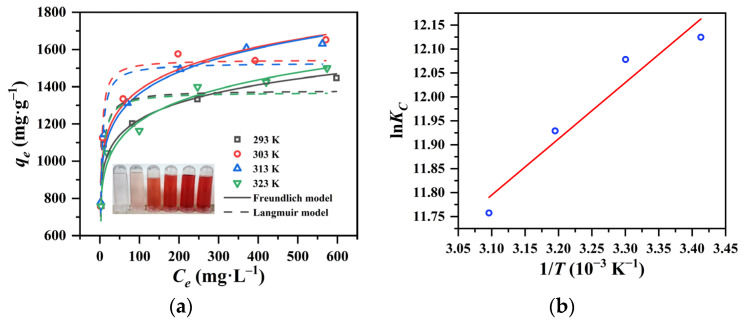
The nonlinear fitting of Langmuir and Freundlich isotherms (**a**) and Van ’t Hoff plot using isotherm constants from the Freundlich isotherm (**b**).

The adsorption isotherm experiments of QAPT-5 for CR were conducted at various temperatures (20, 30, 40, and 50 °C) for 24 h. Figure 9a illustrates the nonlinear results following the Langmuir and Freundlich isotherms. Additionally, Table 4 lists the corresponding fitting parameters. It is evident that the equilibrium adsorption capacity gradually increases with the rise in equilibrium concentration from 2.14 to 624.4 mg∙L^−1^. The adsorption behavior of QAPT-5 towards CR aligns more closely with the Freundlich isotherm at higher temperatures (range from 30 to 50 °C) and with the Langmuir isotherm at low temperatures (20 °C), indicating that heterogeneous surface adsorption occurs at high temperatures and monolayer adsorption at low temperatures. The maximum adsorption capacity ($q_{max}$) of CR displays a decreasing trend at higher temperatures from 30 to 50 °C, with the $q_{max}$ value of QAPT-5 at 30 °C being the largest (1652.2 mg∙g^−1^). Furthermore, thermodynamic studies are crucial for predicting adsorption mechanisms, including physical and chemical processes. The thermodynamic parameters can be calculated based on the Freundlich constant *K_F_* using the following equations [44,45]:

(7) $$K_{C}=\frac{K_{F}\rho }{1000}{\left(\frac{10^{6}}{\rho }\right)}^{\left(1-n\right)},$$

(8) $$∆G^{⊖}=-RT\ln\left(\frac{K_{F}\rho }{1000}{\left(\frac{10^{6}}{\rho }\right)}^{\left(1-n\right)}\right),$$

(9) $$\ln\left(\frac{K_{F}\rho }{1000}{\left(\frac{10^{6}}{\rho }\right)}^{\left(1-n\right)}\right)=\frac{-∆H^{⊖}}{R}\times \frac{1}{T}+\frac{∆S^{⊖}}{R},$$

where *ρ* represents the density of pure water (assumed as ~1.0 g∙mL^−1^), *R* is the universal gas constant (8.3144 J∙(mol∙K)^−1^), and *T* indicates the absolute temperature in Kelvin.

The values of Δ*G*^⊖^, Δ*H*^⊖^, and Δ*S*^⊖^ in Table 5 are consistent with the experimental data of the adsorption isotherms (Figure 9a). The negative Δ*H*^⊖^ indicates the exothermic nature of the adsorption process, leading to a decrease in both the adsorption capacity ($q_{e}$) and the equilibrium constant (*K_C_*) at high temperatures. This exothermic nature suggests the release of energy in the form of heat into the surroundings during the adsorptive process, causing a shift in equilibrium in the opposite direction of the reaction. Additionally, the negative values of Δ*G*^⊖^ at all temperatures suggest that the adsorption phenomenon occurred favorably and spontaneously. As the temperature increases, the more negative values of Δ*G*^⊖^ confirm that the accumulation of CR anions onto QAPT-5 is more favorable at low temperatures. The positive value of Δ*S*^⊖^ suggests an increase in randomness and disorder at the solid/solution interface during the adsorption of CR anions onto QAPT-5. Furthermore, the (+Δ*S*^⊖^) value shows that the adsorption process is entropy-driven rather than enthalpy-driven. The low adsorption enthalpy (Δ*H*^⊖^ < 10 kj∙mol^−1^) supports the idea that the adsorption process is physical adsorption by a weak interaction between the CR and the surface of QAPT-5.

Moreover, Table 6 summarizes the adsorption capacities of various recently reported adsorbents for CR in the literature. Comparatively, the $q_{max}$ of the QAPT-5 in this work is the highest among the investigated adsorbents. That is to say, QAPT-5 exhibits promising performance in the removal of CR from aqueous solutions.

### 3.4. Adsorption Performance of Pellets in a Fixed-Bed Adsorption Column

**Figure 10:**
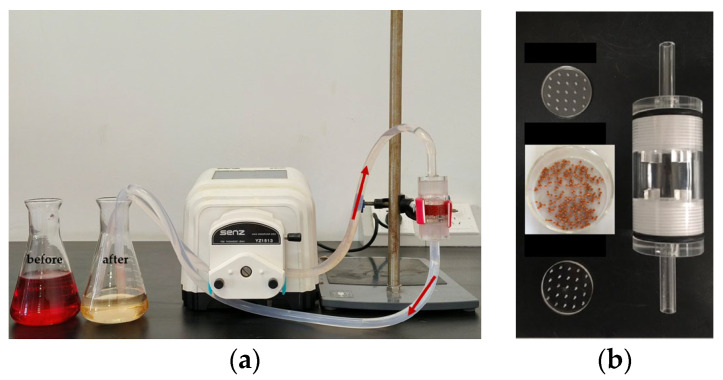
Minor-caliber fixed-bed adsorption column system (**a**) and the adsorption column (**b**).

**Figure 11:**
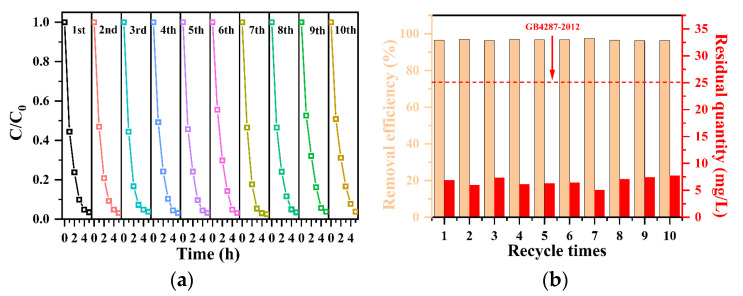
The impact of recycle times (**a**) and removal efficiency (**b**) of the CR solution through the fixed-bed adsorption column of QAPT-5 pellets.

A powdered adsorbent faces significant challenges in practical applications due to the complex and costly separation process to remove the adsorbent from the adsorption system [57]. Therefore, it is crucial to shape the powder into a pellet and utilize a fixed-bed adsorption column [58]. Here, a small-caliber fixed-bed adsorption column was constructed using SA/QAPT pellets, as depicted in Figure 10a. Figure 10b illustrates the adsorption column, which has a length of 65 mm and an inner diameter of 25 mm, filled with approximately 2.2 g of SA/QAPT pellets, including about 2.0 g of QAPT-5 powder. At room temperature (15 °C), a 200 mg∙L^−1^ CR solution was introduced into the adsorption column via an inlet of 200 mL and flowed through the column at a rate of 10 mL∙min^−1^ using a peristaltic pump. The solution then recirculated back to the inlet. The timing of the experiment started when the peristaltic pump was activated, and samples were taken at different intervals to measure the remaining CR concentration using UV-Vis spectroscopy. To assess the recyclability of the fixed-bed adsorption column, the pellets were eluted with ethanol for 12 h. Ten cycles of adsorption–desorption experiments were conducted, with each cycle taking 5 h. Figure 11a demonstrates the impact of the fixed bed cycle time on the concentration of CR in the solution. It is evident that the removal efficiency of a 200 mL solution is approximately 50% after 1 h of cycling. The removal rate increases to 90% after 3 h of recirculation and ultimately surpasses 96% after 5 h of cycling. The removal of CR during a single cycle time exhibits swift removal within the first 3 h, followed by slower removal in the last 2 h. Figure 11b showcases the recyclability of the pellets in the adsorption column after ten adsorption–elution cycles. Even after 10 cycles, the removal rate remains beyond 96% with the concentration of the remaining CR at 7.7 mg∙L^−1^, which is lower than the permitted limit set by the discharge standard (GB4287-2012: the chromaticity is 80 with ca. 25 mg∙L^−1^ CR) [27]. These results highlight that the developed attapulgite is an exceptional adsorbent with practical applications for the removal of CR in aqueous solutions.

Additionally, the production cost of QAPT as an adsorbent material is much lower compared to other materials. The cost of producing 1 ton of QAPT includes approximately CNY 1050 for attapulgite clay, CNY 216 for sodium diphosphate, and CNY 1152 for cetyltrimethylammonium chloride, so the calculated production cost of QAPT is approximately 2418 CNY/t, which is significantly lower than the average market price of commercial adsorbents such as activated carbon (5000 CNY/t) and PPal (3000 CNY/t) [59].

## 4. Discussion

### 4.1. Exploration of Adsorption Mechanism

**Figure 12:**
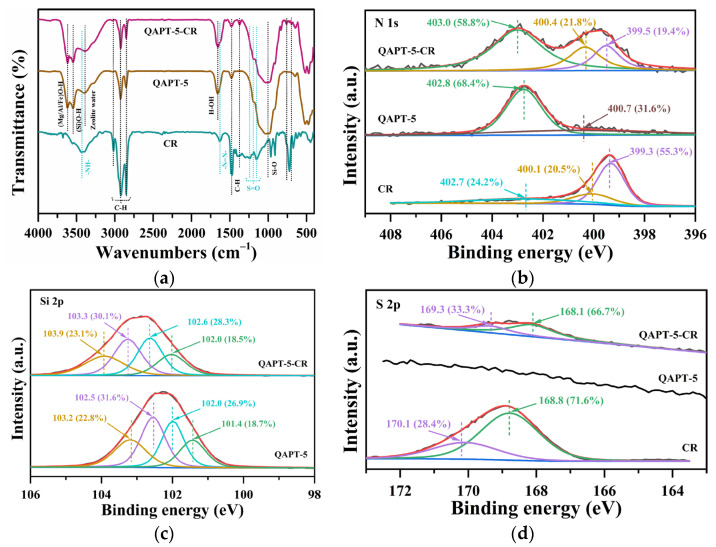
(**a**) FT-IR spectra and XPS spectra of (**b**) N 1s, (**c**) Si 2p, and (**d**) S 2p of QAPT-5 before and after CR adsorption in aqueous solution.

To further investigate the adsorption behavior towards CR, FT-IR and XPS analyses were performed on QAPT-5 before and after the adsorption of CR (QAPT-5-CR) as well as on CR alone. One can observe the stretching vibrations of (Mg/Al/Fe)O-H at 3615 cm^−1^ and (Si)O-H at 3544 cm^−1^ for QAPT-5. Additionally, in Figure 12a, several vibration bands are evident for CR. For instance, peaks at 3433 cm^−1^ and 1630 cm^−1^ are attributed to the stretching vibrations of -NH- and -N=N-, while those at 1241, 1196, and 1151 cm^−1^ correspond to the stretching vibration of S=O [27]. The peaks at 3016, 2926, 2851, and 1476 cm^−1^ are indicative of C-H vibration. Furthermore, the characteristic adsorption band of attapulgite associated with the Si-O-Si bond, which connects two SiO_4_ tetrahedra on a single pyroxene chain, is observed at approximately 1031 cm^−1^. A new vibrational peak emerged around 1370 cm^−1^ in the infrared spectra of QAPT-5 after the adsorption of CR, attributed to the skeletal bending vibrations of CR. Additionally, vibrational absorption peaks within the fingerprint region, ranging from 697 to 758 cm^−1^, were identified as out-of-plane bending vibrational peaks of aromatic hydrocarbons. These results confirm the presence of CR on the attapulgite sample after adsorption.

In order to further investigate the potential interactions between QAPT-5 and CR during the adsorption process, high-resolution XPS spectra of N, Si, and S were carefully collected from the samples before and after adsorption. Interestingly, the peaks of N 1s at 399.5 eV and 400.4 eV for QAPT-5-CR, which are assigned to amine (-NH_2_) and azo (-N=N-), showed a slight shift towards higher binding energies compared with solo CR, as illustrated in Figure 12b [60]. Additionally, the four Si 2p transitions at 101.4, 102.0, 102.5, and 103.2 eV for QAPT-5 slightly moved to 102.0, 102.6, 103.3, and 103.9 eV for QAPT-5-CR in Figure 12c, corresponding to Si(-O)_1_, Si(-O)_2_, Si(-O)_3_, and Si(-O)_4_, respectively [61]. Furthermore, the S 2p spectrum of CR at 168.8 eV (S 2p_3/2_) and 170.1 eV (S 2p_1/2_) displays lower binding energies at 168.1 eV and 169.3 eV for QAPT-5-CR in Figure 12d, indicating the presence of -SO_3_^2−^ from CR [62]. These findings suggest that there may be some interactions occurring between QAPT-5 and CR during the adsorption process, consistent with the thermodynamics analysis.

**Figure 13:**
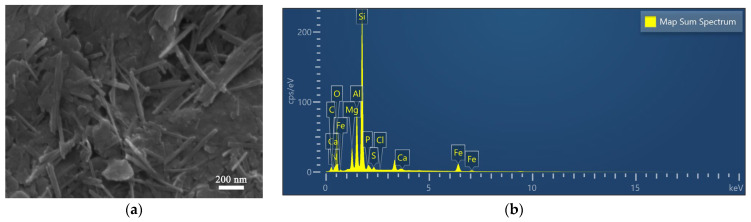
SEM image (**a**) and EDX analysis (**b**) for QAPT-5-CR.

Figure 13 illustrates the SEM micrographs of the sample post-CR adsorption by QAPT-5 along with the outcomes of the EDX analysis. In Figure 13a, it is evident that following CR adsorption, the sample experiences more severe agglomeration and a blocky buildup. Furthermore, the EDX elemental analysis results indicate an increase in the C and S elemental content of sample QAPT-5 post-CR adsorption, attributed to the adsorption of CR onto the sample (Figure 13b).

The zeta potential test results of the adsorbent samples further reveal a gradual increase in zeta potential as the molar mass of CTAC increased (Table 7). The zeta potential of the adsorbent samples changed from negative to positive when the molar mass of CTAC reached 3.0 mmol. The highest zeta potential value of 8.25 mV was recorded at a molar mass of 5.0 mmol. This pattern in zeta potential is found to be consistent with the adsorption capacity of the samples for CR. After the adsorption of CR, the zeta potential of adsorbent QAPT-5 dramatically dropped from 8.25 mV to −24.8 mV. These findings indicate that the adsorption mechanism of CR by the adsorbent QAPT primarily involves electrostatic interactions.

**Figure 14:**
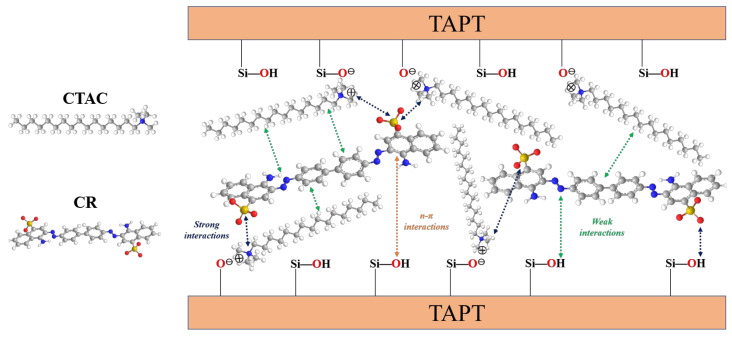
Proposed mechanism for Congo red’s adsorption onto QAPT-5 surface.

In summary, as revealed by IR analysis, the predominant adsorption sites on the surface of QAPT-5 are primarily (Mg/Al/Fe)O-H, (Si)O-H, and quaternary ammonium positive ions. The adsorption mechanism of CR on the QAPT-5 surface primarily involves two types of interactions: strong interactions such as electrostatic attraction and hydrogen bonding, and weak interactions including van der Waals forces and n-π interactions. The zeta potential of QAPT-5 indicates that negatively charged CR molecules are readily attracted to positively charged QAPT-5, representing a pivotal interaction between the dye molecule and the adsorbent surface. The adsorption mechanism also encompasses hydrogen bonding interactions between hydrogen atoms on the surface of QAPT-5 and the SO^3−^ groups within the structure of the CR dye. Additionally, the delocalization of the lone pair of electrons from the O atom to the π-orbital of the dye’s aromatic ring arises from n-π interactions. Collectively, these observations elucidate the adsorption of the CR dye onto the QAPT-5 surface (Figure 14).

### 4.2. Perspectives about Refinement and Application of Low-Grade Attapulgite

While purification technologies for attapulgite have made notable advancements over the years, most efforts have been on refining medium-to-high-grade attapulgite and separating bar crystals. This is because of the complexity of associated mineral species, which presents a major obstacle in purifying low-grade attapulgite, especially impurity minerals that have similar physical and chemical properties to attapulgite, such as muscovite and montmorillonite. Through the process of colloidal mill-assisted ultrasonic-fractional centrifugation, a substantial removal of impurity minerals like quartz and dolomite is achieved, increasing the purity of the refined attapulgite to 60%. However, the content of muscovite, an associated mineral, shows minimal change before and after the purification of the raw ore. Therefore, future endeavors in purifying low-grade attapulgite may focus on addressing associated minerals with similar physical and chemical properties to attapulgite.

## 5. Conclusions

Here, a colloidal mill-assisted ultrasonic-fractional centrifugation method has been developed to purify low-grade attapulgite clay. The refined attapulgite (TAPT) has significantly improved in purity from 16.9% to 60%, specific surface area from 60.57 m^2^ g^−1^ to 135.5 m^2^ g^−1^, and pore volume from 3.42 mL g^−1^ to 17.67 mL g^−1^ in relation to natural ore. Further, TAPT undergoes hydrothermal modification by CTAC from negative charge to positive and then exhibits promising adsorption performance towards Congo red in both types of powder and pellets. The kinetics follow the PSO model, primarily controlled by a multi-step mechanism involving film diffusion, intra-particle diffusion, and adsorption attachment, and the isotherms follow the Langmuir model at 20 °C and the Freundlich model at high temperatures (30, 40, and 50 °C), with the maximum $q_{max}$ of 1652.2 mg∙g^−1^ at 30 °C. Therefore, this work opens a new way to the high-value utilization of low-grade attapulgite clay in practical applications.

## Figures and Tables

**Table 1:** Chemical composition of APT and TAPT.

Samples	SiO_2_/%	MgO/%	Al_2_O_3_/%	Fe_2_O_3_/%	P_2_O_5_/%	CaO/%	K_2_O/%	Na_2_O/%	TiO_2_/%	Other/%
APT	48.74	5.64	14.69	16.63	0.19	4.47	7.18	/	1.35	1.11
TAPT	52.98	7.24	13.95	14.85	1.56	0.73	4.99	1.26	0.36	2.08

**Table 2:** BET analysis results for APT, TAPT, and QAPT adsorbents.

Samples	*S*_BET_ (m^2^/g)	*V*_tot_ (cm^3^/g)	*D*_pore_ (nm)
APT	60.57	0.15	3.42
TAPT	135.5	0.50	17.67
QAPT-0.5	120.4	0.55	17.46
QAPT-1	100.3	0.51	17.62
QAPT-3	72.53	0.41	17.55
QAPT-5	71.29	0.43	17.43

**Table 3:** The fitting results of PFO and PSO adsorption kinetics of TAPT and QAPT samples for CR adsorption.

Samples	$q_{e,\;\exp}$ (mg·g^−1^)	Pseudo-First-Order Model			Pseudo-Second-Order Model		
		$q_{e,\;\mathrm{cal}}$ (mg·g^−1^)	$k_{1}$·(min^−1^)	*R* ^2^	$q_{e,\;\mathrm{cal}}$ (mg·g^−1^)	$k_{2}$·(g·mg^−1^·min^−1^)	*R* ^2^
TAPT	198.8	174.7	4.125 × 10^−1^	0.8342	179.6	3.890 × 10^−3^	0.8870
QAPT-0.5	491.3	437.4	1.362 × 10^−1^	0.8682	457.1	4.217 × 10^−4^	0.9368
QAPT-1	706.2	590.9	1.434 × 10^−1^	0.7820	624.9	2.778 × 10^−4^	0.8709
QAPT-3	752.4	642.1	3.773 × 10^−2^	0.7675	684.3	8.847 × 10^−5^	0.8650
QAPT-5	758.4	671.6	3.476 × 10^−2^	0.7942	693.8	1.223 × 10^−4^	0.8848

**Table 4:** Langmuir and Freundlich isotherm parameters for CR adsorption.

*T*/K	$q_{\max,\;\mathrm{expl}}$/ mg·g^−1^	Langmuir Model			Freundlich Model		
		$q_{\max,\;\mathrm{cal}}$/mg·g^−1^	*K_L_*/L·mg^−1^	*R_L_* ^2^	*K_F_*/(mg·g^−1^)/(mg∙L^−1^)^n^	*n*	*R_F_* ^2^
293	1447.2	1381.4	0.3238	0.9161	761.9	0.1027	0.9080
303	1652.2	1546.3	0.4547	0.9243	825.8	0.1119	0.9307
313	1630.2	1529.0	0.3632	0.8940	786.9	0.1192	0.9615
323	1500.0	1371.3	0.3337	0.8078	690.9	0.1222	0.9776

**Table 5:** Thermodynamic parameters calculated from the Freundlich constant.

*T*(K)	*K_C_*	${∆G}^{⊖}$ (kj/mol)	${∆H}^{⊖}$ (kj/mol)	${∆S}^{⊖}$ (j/(mol∙K))	Van ’t Hoff Equation
293	12.12	−29.63	−9.77	67.77	y = 1175.2x + 8.1516
303	12.08	−30.31			*R*^2^ = 0.9356
313	11.93	−30.98			
323	11.76	−31.66			

**Table 6:** Comparison of the adsorption quantity for CR with different adsorbents.

Adsorbents	$q_{\max}$ (mg/g)	pH Value	Ref.
Organo-attapulgite	189.39	7.0	[46]
TiO_2_/palygorskite	518.13	7.0	[47]
Fe_2_O_3_@CeO_2_-ZrO_2_/Palygorskite	118.75	3.4	[48]
CTA-QS-1.2	664.29 ± 3.92	7~8	[39]
DTA-QS-1.2	684.01 ± 8.50		
CTA-1.2	145.1 ± 3.61		
Pal@C	34.40	1–13	[49]
ACNTs	467.97		
Mg-Al-Fe MMO	1369	13	[50]
Palygorskite/MIL-88A(Fe)	1141.4	10	[51]
APTES/ATP/6	142.93	6.5	[52]
Amino-functionalized SiO_2_-AlOOH	252.53	-	[53]
Ashitaba waste activated carbons	632.1	7	[54]
Hyper-cross-linked resin	280.0	5.01~8.14	[55]
γ-Al_2_O_3_ fibers	781.25	neutral	[56]
Boehmite	1298.3	neutral	[27]
QAPT-5	1652.2	neutral	This work

**Table 7:** Zeta potential of different attapulgite samples.

Sample	Zeta Potential/mV
TAPT	−31.27 ± 0.32
CAPT-0.5	−17.47 ± 0.40
CAPT-1	−16.63 ± 0.31
CAPT-3	5.22 ± 1.51
CAPT-5	8.25 ± 1.56
CAPT-5-CR	−24.8 ±1.95

## Data Availability

Data are contained within the article.

## References

[1] Bradley W.F. (1940). The structural scheme of attapulgite. Am. Mineral..

[2] Chen H., Zhao J., Zhong A.G., Jin Y.X. (2011). Removal capacity and adsorption mechanism of heat-treated palygorskite clay for methylene blue. Chem. Eng. J..

[3] Murray H.H. (2000). Traditional and new applications for kaolin, smectite, and palygorskite: A general overview. Appl. Clay Sci..

[4] Zhou J., Liu N., Li Y., Ma Y.J. (1999). Microscopic Structure Characteristics of Attapulgite. Bull. Chin. Ceram. Soc..

[5] Murray H.H. (2006). Applied clay mineralogy occurrences, processing and application of kaolins, bentonites, palygorskite-sepiolite, and common clay. Dev. Clay Sci..

[6] Chang P.H., Sarkar B. (2021). Mechanistic insights into ethidium bromide removal by palygorskite from contaminated water. J. Environ. Manag..

[7] Kypritidou Z., Argyraki A. (2021). Geochemical interactions in the trace element-soil-clay system of treated contaminated soils by Fe-rich clays. Environ. Geochem. Health.

[8] Rodrigues P.V., Silva F.A.N.G., Pontes F.V.M., Barbato C.N., Teixeira V.G., de Assis T.C., Brandao V.S., Bertolino L.C. (2023). Adsorption of Glyphosate by Palygorskite. Mater. Res.-Ibero-Am. J. Mater..

[9] de Brito Buriti B.M.A., Barsosa M.E., da Silva Buriti J., de Melo Cartaxo J., Ferreira H.S., de Araújo Neves G. (2022). Modification of palygorskite with cationic and nonionic surfactants for use in oil-based drilling fluids. J. Therm. Anal. Calorim..

[10] da Silva R.P., Dantas T.N.D., Barillas J.L.M., Santanna V.C. (2023). The use of organopalygorskite as rheological additive in non-aqueous drilling fluids: Colloidal stability, contact angle, and cutting’s transport ratio. Geoenergy Sci. Eng..

[11] Ramos-Torres W., Borges-Argáez R., Gonzalez-Chi P.I. (2021). Nanostructured chitosan-palygorskite hybrid microspheres for controlled delivery of thymol. Mater. Res. Express.

[12] Fashina B., Deng Y.J. (2022). Smectite, sepiolite, and palygorskite for inactivation of pyocyanin, a biotoxin produced by drug-resistant Pseudomonas aeruginosa. Microporous Mesoporous Mater..

[13] Junior E.D., de Almeida J.M.F., Silva I.D., de Assis M.L.M., Santos L.M.D., Dias E.F., da Silva F.E., Fernandes N.S., da Silva D.R. (2020). Obtaining and Applying Nanohybrid Palygorskite-Rifampicin in the pH-Responsive Release of the Tuberculostatic Drug. Langmuir.

[14] Ramos-Castillo C.M., Sánchez-Ochoa F., González-Sánchez J., Tapia A., Canto G. (2019). Hydrogen physisorption on palygorskite dehydrated channels: A van der Waals density functional study. Int. J. Hydrogen Energy.

[15] Wang S., Ren H.D., Lian W., Zhang X.M., Liu Z.Y., Liu Y., Zhang T.S., Kong L.B., Bai H.C. (2021). Dispersed spherical shell-shaped palygorskite/carbon/polyaniline composites with advanced microwave absorption performances. Powder Technol..

[16] Almeida J.A., Oliveira A.S., Rigoti E., Neto J.C.D., de Alcântara A.C.S., Pergher S.B.C. (2019). Design of solid foams for flame retardant based on bionanocomposites systems. Appl. Clay Sci..

[17] Lv G.C., Liao L.B., Rao W.X., Liu X.H. (2019). Resource Distribution and Application of Attapulgite. Conserv. Util. Miner. Resour..

[18] Zhou J.Y., Cui B.F. (2015). Discussion on genetic types of attapulgite clay deposits in China. East China Geol..

[19] Allouche F., Eloussaief M., Ghrab S., Kallel N. (2020). Clay material of an eocene deposit (khanguet rheouis, tunisia): Identification using geochemical and mineralogical characterization. Clays Clay Miner..

[20] Boudriche L., Chamayou A., Calvet R., Hamdi B., Balard H. (2014). Influence of different dry milling processes on the properties of an attapulgite clay, contribution of inverse gas chromatography. Powder Technol..

[21] Wang S. (2005). Influence of mechanical squeezing on viscidity of attapulgite. China Non-Met. Miner. Ind..

[22] Xu J.X., Zhang J.P., Wang Q., Wang A.Q. (2011). Disaggregation of palygorskite crystal bundles via high-pressure homogenization. Appl. Clay Sci..

[23] Darvishi Z., Morsali A. (2011). Sonochemical preparation of palygorskite nanoparticles. Appl. Clay Sci..

[24] Asamoah E.N., Liu H., Fan X.Y. (2023). Enhanced dechlorination of 2, 4-dichlorophenol using nanorod palygorskite-loaded Fe/Ni nanoparticles: Performance evaluation, influencing factors and action mechanism of Fe and Ni. J. Environ. Technol. Innov..

[25] Zhao Y., Li F., Zhang R., Evans D.G., Duan X. (2002). Preparation of layered double-hydroxide nanomaterials with a uniform crystallite size using a new method involving separate nucleation and aging steps. Chem. Mater..

[26] Guo S.C., Evans D.G., Li D.Q., Duan X. (2009). Experimental and numerical investigation of the precipitation of barium sulfate in a rotating liquid film reactor. AIChE J..

[27] Li Z., He L., Tian W., Huang R., Wang X., Li D., Tang P., Feng Y. (2021). Batch and fixed-bed adsorption behavior of porous boehmite with high percentage of exposed (020) facets and surface area towards Congo red. Inorg. Chem. Front..

[28] Figueiredo V.V., Vianna E.L.F., Lima B.S., Jesus T.C.L., García-Villén F., Bertolino L.C., Spinelli L.S., Viseras C. (2024). Brazilian palygorskite as an alternative to commercial adsorbents for methylene blue: A discussion about composition, morphology and pore profile. Microporous Mesoporous Mater..

[29] Liu Y.X., Zhong H., Li X.R., Bao Z.L., Cheng Z.P., Zhang Y.J., Li C.X. (2022). Fabrication of attapulgite-based dual responsive composite hydrogel and its efficient adsorption for methyl violet. Environ. Technol..

[30] Giustetto R., Wahyudi O. (2011). Sorption of red dyes on palygorskite: Synthesis and stability of red/purple Mayan nanocomposites. Microporous Mesoporous Mater..

[31] Prouzet E., Khani Z., Bertrand M., Tokumoto M., Guyot-Ferreol V., Tranchant J.-F. (2006). An example of integrative chemistry: Combined gelation of boehmite and sodium alginate for the formation of porous beads. Microporous Mesoporous Mater..

[32] Lagergren S. (1898). About the theory of so-called adsorption of soluble substances. K. Sven. Vetensk. Handl..

[33] Blanchard G., Maunaye M., Martin G. (1984). Removal of heavy metals from waters by means of natural zeolites. Water Res..

[34] Weber W.J., Morris J.C. (1963). Kinetics of adsorption on carbon from solution. J. Sanit. Eng. Div..

[35] Langmuir I. (1918). The adsorption of gases on plane surfaces of glass, mica and platinum. J. Am. Chem. Soc..

[36] Freundlich H. (1906). Über die Adsorption in Losungen. Z. Phys. Chem..

[37] Rusmin R., Sarkar B., Biswas B., Churchman J., Liu Y., Naidu R. (2016). Structural, electrokinetic and surface properties of activated palygorskite for environmental application. Appl. Clay Sci..

[38] Mushtaq M., Wasim M., Naeem M.A., Khan M.R., Yue S., Saba H., Hussain T., Siddiqui M.Q., Farooq A., Wei Q.F. (2020). Composite of PLA Nanofiber and Hexadecyl Trimethyl-Ammonium Chloride-Modified Montmorillonite Clay: Fabrication and Morphology. Coatings.

[39] Bhatt A.S., Sakaria P.L., Vasudevan M., Pawar R.R., Sudheesh N., Bajaj H.C., Mody H.M. (2012). Adsorption of an anionic dye from aqueous medium by organoclays: Equilibrium modeling, kinetic and thermodynamic exploration. RSC Adv..

[40] Thommes M., Kaneko K., Neimark A.V., Olivier J.P., Rodriguez-Reinoso F., Rouquerol J., Sing K.S.W. (2015). Physisorption of gases, with special reference to the evaluation of surface area and pore size distribution (IUPAC Technical Report). Pure Appl. Chem..

[41] Buapuean T., Jarudilokkul S. (2020). Synthesis of Mesoporous Zn-doped TiO_2_ Nanoparticles by Colloidal Emulsion Aphrons and Their Use for Dye-sensitized Solar Cells. Russ. J. Appl. Chem..

[42] Oliveira M.E.R., Santos L.D., da Silva M.L.D., da Cunha H.N., da Silva E.C., Leite C.M.D. (2015). Preparation and characterization of composite polyaniline/poly(vinyl alcohol)/palygorskite. Microporous Mesoporous Mater..

[43] Walter W.J. (1984). Evolution of a technology. J. Environ. Eng..

[44] Ghosal P.S., Gupta A.K. (2015). An insight into thermodynamics of adsorptive removal of fluoride by calcined Ca-Al-(NO_3_) layered double hydroxide. RSC Adv..

[45] Tran H.N., You S.J., Chao H.P. (2016). Thermodynamic parameters of cadmium adsorption onto orange peel calculated from various methods: A comparison study. J. Environ. Chem. Eng..

[46] Chen H., Zhao J. (2009). Adsorption study for removal of Congo red anionic dye using organo-attapulgite. Adsorption.

[47] Peng Y.G., Chen D.J., Ji J.L., Kong Y., Wan H.X., Yao C. (2013). The preparation of titanium dioxide/palygorskite composite and its application in the adsorption of congo red. Environ. Prog. Sustain. Energy.

[48] Ouyang J., Zhao Z., Suib S.L., Yang H.M. (2019). Degradation of Congo Red dye by a Fe_2_O_3_@CeO_2_-ZrO_2_/Palygorskite composite catalyst: Synergetic effects of Fe_2_O_3_. J. Colloid Interface Sci..

[49] Zhong L.F., Tang A.D., Yan P., Wang J.J., Wang Q.J., Wen X., Cui Y. (2019). Palygorskite-template amorphous carbon nanotubes as a superior adsorbent for removal of dyes from aqueous solutions. J. Colloid Interface Sci..

[50] Lu Y.S., Duan F.Z., Zhu Y.F., Wang A.Q. (2023). Sustainable utilization metal ions of acid leaching clay wastewater to fabricate adsorbents for high-efficient removing Congo red and Methyl violet. Chem. Eng. Res. Des..

[51] Gao W.T., Mu B., Zhu Y.F., Wang A.Q. (2023). Preparation of palygorskite/MIL-88A(Fe) composites for high-efficient removal of Congo red. Appl. Clay Sci..

[52] Yang S., Zhao F., Sang Q.Q., Zhang Y., Chang L., Huang D.J., Mu B. (2021). Investigation of 3-aminopropyltriethoxysilane modifying attapulgite for Congo red removal: Mechanisms and site energy distribution. Powder Technol..

[53] Zhang Y.X., Ye Y.J., Zhou X.B., Liu Z.L., Zhu G.P., Li D.C., Li X.H. (2016). Monodispersed hollow aluminosilica microsphere@hierarchical γ-AlOOH deposited with or without Fe(OH)_3_ nanoparticles for efficient adsorption of organic pollutants. J. Mater. Chem. A.

[54] Li Z.C., Hanafy H., Zhang L., Sellaoui L., Netto M.S., Oliveira M.L.S., Seliem M.K., Dotto G.L., Bonilla-Petriciolet A., Li Q. (2020). Adsorption of congo red and methylene blue dyes on an ashitaba waste and a walnut shell-based activated carbon from aqueous solutions: Experiments, characterization and physical interpretations. Chem. Eng. J..

[55] Waheed A., Mansha M., Kazi I.W., Ullah N. (2019). Synthesis of a novel 3,5-diacrylamidobenzoic acid based hyper-cross-linked resin for the efficient adsorption of Congo Red and Rhodamine B. J. Hazard. Mater..

[56] Wang Y., Li W., Jiao X., Chen D. (2013). Electrospinning preparation and adsorption properties of mesoporous alumina fibers. J. Mater. Chem. A.

[57] Jo J.Y., Choi J.H., Tsang Y.F., Baek K. (2021). Pelletized adsorbent of alum sludge and bentonite for removal of arsenic. Environ. Pollut..

[58] Condurache B.C., Cojocaru C., Bargan A., Samoila P., Harabagiu V. (2024). Dynamic Adsorption of a Cationic Dye onto Wool Fibers as Column-Filling Media: Response Surface Optimization and Fixed-Bed Adsorption Modeling. Materials.

[59] Xu C., Feng Y., Li H., Li Y., Yao Y. (2024). Purification of natural palygorskite clay: Process optimization, cleaner production, mineral characterization, and decolorization performance. Appl. Clay Sci..

[60] Chen Y.Y., Yu S.H., Jiang H.F., Yao Q.Z., Fu S.Q., Zhou G.T. (2018). Performance and mechanism of simultaneous removal of Cd(II) and Congo red from aqueous solution by hierarchical vaterite spherulites. Appl. Surf. Sci..

[61] Alexander M.R., Short R.D., Jones F.R., Michaeli W., Blomfield C.J. (1999). A study of HMDSO/O_2_ plasma deposits using a high-sensitivity and -energy resolution XPS instrument: Curve fitting of the Si 2p core level. Appl. Surf. Sci..

[62] Aoopngan C., Nonkumwong J., Phumying S., Promjantuek W., Maensiri S., Noisa P., Pinitsoontorn S., Ananta S., Srisombat L. (2019). Amine-functionalized and hydroxyl-functionalized magnesium ferrite nanoparticles for Congo red adsorption. ACS Appl. Nano Mater..

